# Large Scale Application of Neural Network Based Semantic Role Labeling for Automated Relation Extraction from Biomedical Texts

**DOI:** 10.1371/journal.pone.0006393

**Published:** 2009-07-28

**Authors:** Thorsten Barnickel, Jason Weston, Ronan Collobert, Hans-Werner Mewes, Volker Stümpflen

**Affiliations:** 1 Helmholtz Zentrum München - German Research Center for Environmental Health, Institute of Bioinformatics and Systems Biology (MIPS), Neuherberg, Germany; 2 NEC Laboratories America, Inc., Princeton, New Jersey, United States of America; 3 Chair of Genome-Oriented Bioinformatics, Technische Universität München, Life and Food Science Center Weihenstephan, Freising-Weihenstephan, Germany; Center for Genomic Regulation, Spain

## Abstract

To reduce the increasing amount of time spent on literature search in the life sciences, several methods for automated knowledge extraction have been developed. Co-occurrence based approaches can deal with large text corpora like MEDLINE in an acceptable time but are not able to extract any specific type of semantic relation. Semantic relation extraction methods based on syntax trees, on the other hand, are computationally expensive and the interpretation of the generated trees is difficult. Several natural language processing (NLP) approaches for the biomedical domain exist focusing specifically on the detection of a limited set of relation types. For systems biology, generic approaches for the detection of a multitude of relation types which in addition are able to process large text corpora are needed but the number of systems meeting both requirements is very limited. We introduce the use of SENNA (“Semantic Extraction using a Neural Network Architecture”), a fast and accurate neural network based Semantic Role Labeling (SRL) program, for the large scale extraction of semantic relations from the biomedical literature. A comparison of processing times of SENNA and other SRL systems or syntactical parsers used in the biomedical domain revealed that SENNA is the fastest Proposition Bank (PropBank) conforming SRL program currently available. 89 million biomedical sentences were tagged with SENNA on a 100 node cluster within three days. The accuracy of the presented relation extraction approach was evaluated on two test sets of annotated sentences resulting in precision/recall values of 0.71/0.43. We show that the accuracy as well as processing speed of the proposed semantic relation extraction approach is sufficient for its large scale application on biomedical text. The proposed approach is highly generalizable regarding the supported relation types and appears to be especially suited for general-purpose, broad-scale text mining systems. The presented approach bridges the gap between fast, cooccurrence-based approaches lacking semantic relations and highly specialized and computationally demanding NLP approaches.

## Introduction

The rapidly increasing amount of biomedical publications is a key resource for the automated extraction and inference of relations between biomedical concepts such as protein-protein interactions or regulatory interrelations. Cooccurrence based text mining (TM) applications such as iHOP [1] or EBIMed [2] are a valuable help for extracting related biomedical entities from literature automatically. Applications using cooccurrence usually show an excellent recall rate in combination with a high processing speed which makes them suitable especially for text mining systems covering large text corpora such as MEDLINE. By the nature of this approach, it is not possible to extract the direction and type of the relation between detected cooccurring entities.

In addition to cooccurrence-based approaches, various sophisticated rule-based or machine-learning- approaches considering additional, usually syntactical information of a sentence have been developed to specifically extract relations of a certain semantic type. Commonly, syntax trees are generated and the tree constituents are semantically classified in a subsequent processing step. Both tasks can be computationally expensive. In addition, syntax trees are not easy to interpret for non-linguists and the development of rules and regular expressions for searching those trees or, in case of machine-learning based approaches, annotating a training corpus of sufficient size, are time consuming tasks.

**Figure 1:**
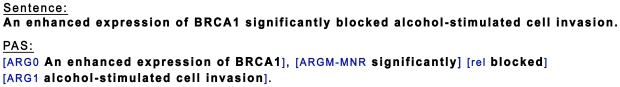
Biomedical sentence and one corresponding PAS for the verb ‘blocked’. A sentence annotated by SENNA with semantic, PropBank conforming roles. ARG0: The subject phrase of the verb/the blocker; ARG1: the argument role of the verb, the thing blocked; rel: the verb; ARGM-MNR: modifier argument for manner.

A comparatively new approach belonging to the second application type is Semantic Role Labeling (SRL). SRL determines the semantic roles syntactic constituents of a sentence play in relation to a certain predicate. A set of a verb and its corresponding semantic arguments is called a “predicate-argument-structure” (PAS) (figure 1). Typical semantic roles according to the annotation system of the PropBank corpus [3], a newspaper corpus many of the existing SRL systems are based on, are the subject role (labeled with “ARG0”) and the argument role (typically “ARG1”) of a verb. The semantic roles of the remaining three core arguments ARG2-ARG5 are more diverse and depend on the verb of each PAS [3]. All verbs can also have so-called “modifier” arguments (ARGM) such as location (“ARGM_LOC”), time, cause and others.

The application of Semantic Role Labeling in the biomedical domain was conceptually introduced as a promising approach by Kogan Y. et al. [4], who showed that 76% of the verbs used in biomedical literature are also contained in the PropBank corpus. Until recently, many SRL systems were based on the assumption that the accurate extraction of semantic roles contained in natural language texts requires the knowledge of the syntactical structure of its sentences. Constituents in a parse tree of a sentence were assigned to a predefined set of semantic roles using a classifier, e.g. support vector machines (SVM), decision trees or log linear models. The generation of syntax trees and in many cases also the classification step are demanding, time consuming processes. Most SRL systems or full syntactical parsers used to derive semantic roles require on average 1–3 seconds to calculate the syntactical structure of a sentence (e.g. Lexicalized Stanford Parser [5] used by the RelEx system [6]: ∼1–14 seconds/sentence, Charniak-Lease Parser [7], an adaptation of the Charniak Parser [8] to biomedical text: 2.7 seconds [9], BIOSMILE SRL system [10], [11]: 1,99 seconds/sentence (according to the authors) although meanwhile also faster alternatives exist (Enju 2.3 parser [12]: less than 50–500 ms/sentence [13]). SENNA [14], [15], a semantic role labeling program trained on the PropBank corpus, does not rely on the extraction of syntax trees for assigning semantic roles to sentence constituents. Instead, it uses a radically different approach compared to the existing SRL programs: skipping the step of syntax tree generation, SENNA's neural network architecture was trained directly on some basic, quickly derivable sentence features. SENNAs output is a sentence annotated with PropBank arguments delivering semantic roles like subject, argument, negation, location, manner and others. Depending on the sentence length, the parsing speed of SENNA is between 25 and 390 ms/sentence.

SRL by itself does not deliver subject – argument relationships between entities but delivers phrases and sentence fragments fulfilling a certain semantic role. Biological entities, e.g. proteins, mentioned within the ARG0 part of a sentence will here be referred to as “actors”, those mentioned within ARG1 parts “targets”. In order to assess the applicability of SRL for extracting relations between biomedical entities, we examined how often the simplifying assumption holds true that all entities in the ARG0/ARG1 parts generated by a SRL program indeed act as actor/target in the sense of the verb. This question is of crucial importance to assess whether the proposed SRL based approach can be used with sufficient reliability to build up a large scale biomedical text mining system. SENNA combines high processing speed with high semantic labeling accuracy and, in contrast to the high-speed (“mogura”) version of Enju, also tags modifier arguments in addition to the core arguments ARG0-5. Therefore, we choose SENNA for the evaluation of SRL based relation extraction (RE), applied SENNA to almost 90 million MEDLINE sentences and also compared its speed with syntactic parsers commonly used for relation extraction in the biological domain.

## Methods

For PAS generation, the neural network based SRL system “SENNA” [14], [15] was applied. SENNA is the fastest highly accurate SRL program available today. Currently, there exist two variants of the program: SENNA*, the variant we used for PAS generation and performance evaluation, and SENNA-web, the latest implementation downloadable from the NEC-labs website. The processing times reported in the following section differ from those published by Collobert R. and Weston J. 2007 because the SENNA implementations used for this analysis include some text pre-processing functions which were not included in [14].

### SENNA Algorithm

SENNA is a deep convolutional neural network architecture designed specifically for the task of semantic role labeling. For this work we used a variant of the algorithm described in [15] employing some additional text pre-processing steps. The algorithm at a conceptual level takes a sentence as input and outputs semantic roles for each word in the input sentence for every identified verb. The network is trained using the PropBank database which is a set of sentences from the Wall Street Journal which have been manually annotated with semantic tags. The PropBank formalism is that each verb (and verb sense) has a human annotated frame which indicates the typical roles that should be assigned for this verb.

The first task of SENNA is to locate the verbs in the input sentence. This is achieved by training a part-of-speech tagger, and applying it at test time. SENNA then outputs a role for a chosen word in the input sentence given the verb of interest. Hence, the SENNA architecture is thus applied (number of verbs) * (sentence size) times. The internal structure of this architecture is as follows: words are first represented via a binary encoding as vectors of dimension (dictionary size), i.e. these vectors are all zeros apart from one 1, indicating the word's index into the dictionary. In the first layer of the network these vectors are multiplied by a (dictionary size) x (50) dimensional weight matrix to yield 50-dimensional feature vector representations for each word (this is often called a “Lookup Table” layer). This weight matrix is learnt as part of the backpropagation step of the neural network, and embeds words in a low dimensional feature space that the network can use to represent syntactic and semantic features relevant for the task. Extra features are also added to encode for each word whether it is the verb of interest or the word to be tagged. The next layer applies a convolution, i.e. a sliding window with a window size of 3 words across the sentence, that outputs 200 features for each position of the window to the next layer. This layer finds *local* features amongst neighboring words. The next layer applies a max operation across the sentence length to find *globally* relevant parts of the sentence for the classification task at hand. The final layers are classical linear layers, outputting for the word of interest which of the 23 classes of semantic roles it should be assigned (including the case of no role at all). On the PropBank test set SENNA has a per word error rate of approximately 14.5% which is competitive with other state-of-the-art methods, e.g. the (SVM-based) ASSERT parser [16]. For a more detailed description of the algorithm and the evaluation process please see [14], [15]. SENNA is available at: http://ml.nec-labs.com/software.php?project=senna.

### PAS Generation

In order to assess the applicability of SENNA for relation extraction regarding speed and reliability on biomedical texts, 89 million sentences were derived from 17 million MEDLINE citations, 44.000 Open Access PubMed Central (PMC) full text articles and 17.000 OMIM records. Based on this set of sentences, 78 million PAS structures containing at least one ARG0 and ARG1 role were extracted.

### Dataset for Evaluating the Relation Extraction step

In order to evaluate the accuracy of the assumption that the relation between sentence parts labeled with “ARG0” and “ARG1” can be transferred upon the biological entities mentioned therein, two datasets were used: the first dataset (LLL'05) consisted of 77 sentences (genic_interaction_data.txt and genic_interaction_data_coref.txt) provided as training data set in the LLL 2005 Genic Interaction Extraction Challenge [17] (data.jouy.inra.fr/unites/mig/text/LLLChalenge05/data/train/task1/). Each sentence had been annotated with at least one interaction consisting of actor, target and the direction of the relation. The exact type of the relationship was not provided but was annotated manually for each sentence from the provided interaction information during the evaluation process. The data set did not contain any negative example sentences. The second dataset (BC-PPI) consisted of 173 sentences with at least one annotated relation and 743 negative example sentences. The sentences had been extracted from the BioCreAtIvE 1A task dataset [18] by randomly selecting 1000 sentences and manually adding annotations on protein names and protein-protein interactions (see www2.informatik.hu-berlin.de/∼hakenber/corpora/). Each annotated PPI consisted of an actor, a target and a specified relation connecting both entities.

The chance of a falsely detected relation is assumed to rise with increasing length and complexity of a sentence and its syntactical components (e.g. by inclusion of one or more nominalizations or conditional phrases which may invert the meaning of a PAS). Therefore we checked whether the average length of the sentences in the test sets resembles the length of an average sentence taken from MEDLINE, OMIM or PubMed Central. We found that the average sentence length of both test data sets resemble the length of sentences contained in the most relevant biomedical literature sources (table 1). Thus, we assume that the accuracy obtained from the test data should be similar to the accuracy obtained when applying SENNA to large literature sources.

**Table 1:** Determination of the average sentence length in the test sets as well as in the three sources of biomedical literature used for PAS extraction.

source	# evaluated sent.*	avg. sent. length*	avg. sent. length**
MEDLINE	84.930.500	137	152
PMC	3.523.463	207	208
OMIM	434.567	-	146
BC-PPI	1000	150	
LLL'05	77	172	

Evaluation of average sentence length in characters for different literature resources in the biomedical domain.

*) including titles.

**) excluding titles.

## Results and Discussion

We used SENNA to extract 78 million PASs containing at least one ARG0 (subject role) and one ARG1 (argument role) component from 89 million biomedical sentences. The processing time strongly depends on the number of words within a sentence. We observed processing times of ∼25 milliseconds/sentence for sentences containing ∼70 characters and 390 milliseconds/sentence for sentence length of ∼240 characters. The whole set of 78 million PASs could be generated on a 104 node Linux cluster within 3 days. By confining the PAS generation to sentences with a high chance of mentioning a relation (e.g. those containing at least two biomedical entities), even more restricted hardware resources should be able to parse the most relevant sentences of MEDLINE within a few days time.

In addition, we calculated PASs for sentences of the 2005 LLL Challenge training data set as well as for the BioCreAtIvE - PPI dataset to examine the applicability of the hypotheses that entities mentioned within a sentence component labeled as subject phrase (ARG0) or argument phrase (ARG1) indeed act as biological “actor”/“target” in the way implied by the corresponding predicate of the PAS.

### Performance

To evaluate SENNA's performance, four sets of 500 sentences with different ranges of length were processed on an Intel Dual Core Pentium processor with 2.40 GHz and 4 GB memory. In addition to the SENNA variant we used for generating the PASs (SENNA*) we also tested an even faster SENNA variant downloadable from the NEC labs site (SENNA 1.0 web).

**Figure 2:**
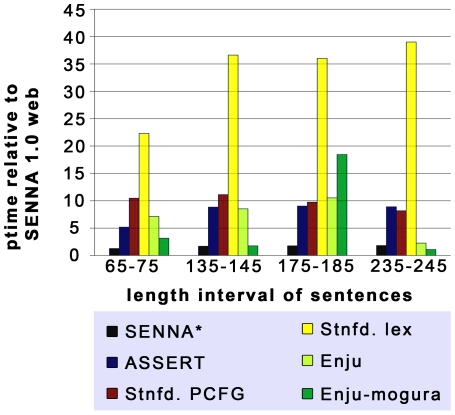
Performance comparison of SENNA with common SRL programs and syntactic parsers. Performance time (ptime) of SENNA* (the SENNA variant we used for PAS generation), ASSERT and Stanford PCFG and lexicalized parser were measured relative to SENNA 1.0 web version on four test sets of 500 sentences each. The length interval of the sentences ranged from 65–75 characters for the first test set to 235–245 characters in the fourth test set. The Enju-mogura parser appeared to have difficulties specifically with the 175 character test set we used, processing times on other sentences of similar sentence length resulted in processing times comparable to the mogura results on the 65, 135 and 235 characters test sets.

As figure 2 shows, SENNA 1.0 web outperforms ASSERT by a factor of 5–10. For SENNA*,

the performance gain ranges between 4 and 8. The performance gain of SENNA 1.0 web compared to ASSERT is largest (factor 11) at a sentence length typical for biomedical sentences derived from MEDLINE abstracts (∼140 characters) and even more impressing if compared to slower parsers such as the lexicalized Stanford- or Bikel parser. The processing times of Enju were similar to those of ASSERT while its high speed variant reached processing times similar to those of SENNA*. In absolute numbers, the processing speed of the SENNA 1.0 web/SENNA* versions ranged from 20 ms/25 ms per sentence from the shortest sentence set (65–75 characters) to about 220 ms/394 ms per sentence for the longest sentence test set. The processing time of the Bikel Parser was very long (612–42000 ms per sentence) and was therefore not included in the figure.

### Verb count

Within those 78 million PASs, 180.000 different terms were labeled by SENNA as verbs, here referred to as “verb-candidates”. A list of verb-candidates for all generated PASs was extracted and can be downloaded from our website (ftp://ftpmips.helmholtz-muenchen.de/textmining). The verb-candidates are ordered in descending occurrence number and may help in the development of biomedical text mining systems, especially in the compilation of verb sets expressing a certain relation type specific for the biomedical domain. The 5000 most frequently occurring verbs-candidates were manually evaluated to detect words wrongly classified as verbs. The fraction of wrongly assigned verb-candidates was determined by examining sub-sets of 50 verb-candidates each.

**Figure 3:**
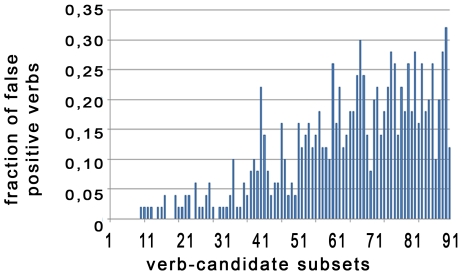
Histogram of the fraction of wrongly predicted verbs covering the 5000 most frequent verb-candidates. After checking those 5000 verb-candidates manually for false verb assignments, the candidates were grouped in 100 subsets of 50 verb-candidates. For each group the fraction of verb-candidates wrongly labeled as “verb” by SENNA was evaluated (y-axis). The histogram shows these 100 subsets ordered by descending candidate – frequency from left to right. With decreasing term frequency, the number of wrong assignment rises.

Figure 3 illustrates the most frequent 450 verb-candidates being all correctly labeled as verb. With decreasing verb-frequency, the fraction of wrong assignments per window slowly increases reaching ∼32% for the verb-candidates 4900–4950. The large majority of verb-candidates with a count less than three within the extracted PAS corpus were erroneously labeled as predicates. The main source of wrong assignments originated from classifying domain-specific terms like metabolite and gene names as verbs. We observed also some problems in the detection of compound verbs like “(LPS)-stimulated” leading to verbs starting with a hyphen (“-stimulated”).

We did not find a threshold below which all verb-candidates could reliably be classified as erroneous: each verb usually occurred in multiple verb forms of different frequencies, e.g. reattach (count = 97), reattached (count = 91), reattaching (count = 63), reattaches (count = 4). Even verb-candidates with a very low total count may therefore be connected to a meaningful relation.

In practice, the large number of verb-candidates falsely classified as verb does not affect the relation extraction step negatively. For building up an relation extraction system based on PASs, a positive-list of verbs resembling a certain relationship like “activation” or “inhibition” has to be created first, and only PASs containing one of those verbs should be considered. This constraint can also help to reduce the amount of data that has to be handled. The modeling of semantic relation types by assigning sets of verbs to each relation type renders this approach as highly generalizable and flexible. Relation types like “regulation” may cover a broad set of verbs while the list of verbs describing “phosphorylation” events will be comparatively small. For the purpose of relation type modeling, the generated verb list can be of high value. The list may assist in the development of predicate sets resembling a certain relation type that covers the most frequent verb forms used in the biomedical literature.

### Relation Extraction

SRL systems label sentence parts or words according to their semantic roles in relation to the verb. Roles can be subject, argument, localization or similar types. The aim of RE in the biomedical domain is the extraction of relations not between phrases but between biological entities such as miRNA/gene, enzyme/metabolite or gene/disease. An important question for the use of SRL systems for relation extraction without further post-processing is the validity of the simplifying assumption that all entities detected within a phrase of a certain semantic role really act in the same way as the sentence part they are embedded in. For example, in the sentence “RbAp46 gene activates the expression of IGFBP-rP1 gene in K562 leukemic cells”, the gene RbAp46 within the ARG0 phrase “RbAp46 gene” indeed acts as the “actor” on the “target” gene IGFBP-rP1. This assumption is incorrect for sentences like “The inhibition of RbAp46 gene activates the expression of IGFBP-rP1 gene in K562 leukemic cells”. The relation stated within the second (fictional) sentence actually is “RbAp46 inhibits IGFBP-rP1”.

We evaluated the accuracy of relation extraction based on the PASs generated by SENNA on the sentence level by using two different test datasets, the LLL'05 training- and BC-PPI corpus. For each dataset, all PASs were calculated by SENNA. Only those PASs were considered in the evaluation which contained at least one ARG0 and one ARG1 label and a biological meaningful verb like “activates”, “binds” or “inhibits”. For example, PASs like “[Analysis of the expression of a translational ywhE-lacZ fusion] (ARG0) [showed] (verb) [that ywhE expression is sporulation specific] (ARG1) ” were ignored as verbs like “suggest”, “show” or “proved” occur frequently in biomedical text but usually do not contain relevant information (the ARG0 part of such PASs in most cases lacks a biomedical entity).

For all annotated interactions in both test corpora, a detected relation was considered as true positive (TP), if the actor was mentioned within the ARG0 part, the target was mentioned within the ARG1 part and the verb of the corresponding PAS resembled the meaning of the annotated relation. If no such PAS existed for the sentence in which the interaction was annotated, this observation was considered as false negative (FN). If the actor was mentioned within the ARG1 part and the target in the ARG0 part or if any additional PAS existed for this sentence wrongly connecting the entities annotated within this sentence by a biological meaningful verb (e.g. “activates”, “binds”, “interacts” etc.), this observation was counted as false positive (FP). Some of the sentences of the BC-PPI dataset contained relations between proteins which had not been annotated in the corpus for not being a protein-protein interaction in the strict sense. Those relations were additionally taken into account in the evaluation. As the focus of our examination was the RE step, not Named Entity Recognition (NER), the problem of detecting proteins and genes in the test sentences was not addressed. Rather, the annotated gene- and protein names were used as provided by the LLL'05 and PPI datasets and precision and recall values therefore resemble only the accuracy of the relation extraction step.

As table 2 shows, the precision P of relation extraction in most cases delivered reliable results (78% on the LLL'05 and 68% on the BC-PPI data set) while the recall R (38% and 45%) was comparatively low resulting in an F-measure of 0.51 and 0.55. It should be possible to raise the observed recall by considering additional frames and arguments like ARG0-ARGM_LOC in addition to the evaluated ARG0-ARG1 relations. As has been shown previously for the BIOSMILE system [11], a further gain in precision as well as recall may be achieved by retraining the neural net on a biomedical text corpus. We expect that the effect is small. In most cases, the FN results of our analysis were caused by relations expressed within nominalizations or complex syntactical expressions, which also occur in newspaper texts SENNA was trained on. The fraction of biomedical proper nouns erroneously labeled as verb would be reduced by a retraining on biomedical sentences. As we used a positive list of relevant verbs, this reduction regarding the number of generated PASs would help to reduce the amount of data to be managed but would not improve the accuracy of the proposed approach.

**Table 2:** Evaluation of SENNA* on LLL'05 and BC-PPI corpus.

Test sets	TP	FP	FN	TN	Prec.	Rec.	F-measure
LLL'05	63	18	103	-	0.78	0.38	0.51
BC-PPI	133	62	159	799	0.68	0.45	0.55
Total	196	80	262	799	0.71	0.43	0.54

Precision and recall of RE step applied on the LLL'05 and BC-PPI data set. All TPs, FPs and FN in both data sets were summed up for an overall value for precision, recall and F-measure.

The comparison between the proposed approach and current, publicly available text mining systems is difficult since precision and recall values of these systems include losses caused by the NER step. In addition, the observed accuracies strongly depended on the test set of sentences or interaction data the published text mining tools have been tested on. The RelEx system[6], a text mining system based on dependency parse trees and rules, reached the highest F-measure of all competing systems in the LLL challenge. On the LLL training corpus which was also used for our evaluation, Fundel K. et al. (2006) reported precision/recall (P/R) values of 68%/83% (RE and NER) for predicting the entity position and direction of the relations mentioned in the corpus.

Precision of our approach on the sentence level (78% on the LLL training corpus, 68% on the BC-PPI corpus) is in the same range as that of the RelEx system (68% on the LLL training corpus) although it has to be noted that the precision given for RelEx comprises also errors introduced by the NER step which were not considered in our purely RE based examination. The recall on the sentence level of our approach is comparatively low. However, due to the substantially increased processing speed of SENNA compared to existing syntax tree based approaches, very large text resources such as the whole MEDLINE database can be processed within only a few days time which is not an option for many of the existing text mining systems having processing times of several seconds per sentence.

Considering the fact that many relations between biomedical entities are described multiple times in MEDLINE abstracts, we expected that the recall of our approach at the relation level is significantly higher than the observed 43% on the sentence level. In order to check this assumption, a maximum of 50 direct or indirect, experimentally verified regulatory relations of four breast cancer related proteins (BRCA1, BRCA2, CXCR4, PTHrP) were extracted from the STRING database and considered as gold standard. The relations contained in this gold standard were compared to the predictions generated by the proposed SRL based approach: we compiled a dictionary of gene/protein names from EntrezGene and SwissProt and located the positions of those names within the ARG0 and ARG1 parts of 78 million PASs with the help of the Java Lucene text search- and indexing API. A list of verbs expressing a direct or indirect relationship was compiled manually which can also be downloaded from the ftp server hosting the test results. A regulatory relation between two proteins A and B was postulated in our approach if the ARG0 part of at least one PAS with “regulatory verb” mentioned protein A in the ARG0 part and protein B in the ARG1 part. The relevance/confidence value of the extracted relations between A and B depended on the number of PASs supporting a relation. The more regulatory PASs mentioning A in ARG0 and B in ARB1, the higher the confidence score.

We examined two questions. First, we wanted to know which fraction of the relations contained in the gold standard derived from STRING could be detected with our proposed approach. Second, we wanted to find out, how many false positive predictions the SRL based RE approach would generate within the first 50 predicted relations with the highest confidence value/the highest number of supporting PASs. The details of this subject-based accuracy evaluation can be found on our ftp site. In summary, 49 out of 80 gold standard relations (61%) could be retrieved. At least for this small test set of four proteins, the relation-based recall value is notably higher than the recall on the sentence level. Regarding the second question, 158 out of the 200 predictions with the highest confidence value (50 predictions for each of the four proteins) were true positive predictions (79%).

While the cited text mining system was developed specifically for the task of extracting interactions between a limited set of entity types such as genes or proteins, the proposed approach follows a general scope as it connects any type of entity mentioned within the ARG0 or ARG1 sentence part semantically depending on the verb of the corresponding PAS. As often the case, the general applicability of an approach goes at the expense of accuracy. However, our results are favorable for the development of a large scale text mining system based on SRL.

The evaluation results, verb lists and supplementary information can be downloaded from ftp://ftpmips.helmholtz-muenchen.de/textmining.

### Conclusions

We presented the use of a novel, SRL (SENNA) based approach for fast and reliable semantic role labeling of biomedical text corpora. The presented RE approach is well suited to detect relations expressed as predicate – argument structures while the detection of relations expressed within nominalizations and syntactically more complex relational expressions remains a challenge. In order to extract relations from specialized biomedical domains, tailored solutions including rule- and regular expression-based approaches adapted to the syntactical peculiarities of the sub-domain specific literature will be the methods of choice. Such a case, for instance, could be the extraction of protein transport relations mentioned within GeneRIFs, a set of sentences in the Entrez Gene database describing the function of a gene, where 85% of the protein transport predicates were reported to be used as nouns [22]. For the construction of a general purpose text mining system covering a broad range of entity- and relation types, on the other hand, we think that SRL based relation extraction using SENNA or an adaptation of SENNA to the biomedical domain is an appropriate approach for the following reasons:

1. SENNA is not constrained to a limited set of 30 verbs like the BIOSMILE system but covers the vast majority of verbs used in the biomedical domain.
2. Although trained on the newspaper text corpus PropBank, the presented RE approach based on PAS structures generated by SENNA displayed good precision and acceptable recall values. In the future, recall could be improved by retraining SENNA on a large biomedical text corpus and by considering relations expressed within additional frames and semantic roles like ARG2 or ARGM_LOC. Due to the redundancy of interaction information contained in literature, we expect that the recall rate of a large scale SENNA based text mining (TM) system is significantly higher on the relation level than the observed 43% on the sentence level.
3. In contrast to the wide-spread syntax-tree based approaches, the SRL based RE approach has a significant advantage by avoiding domain-specific rules or regular expressions for the extraction of relations between biomedical entities. The construction of rules is a time consuming process, especially if the constructed relation extraction system has to cover many different types of relations between multitudes of different biological entities. Rule development requires detailed knowledge of the output generated by the individual parser. This output depends on singular or plural forms, the tense, active or passive voice of the sentence as well as on peculiarities of the syntactical structures preferred by authors in various biomedical sub-domains. The semantic PropBank tags generated by SENNA, on the other hand, can easily be interpreted without deeper linguistic knowledge and used for a PAS-based relation extraction between biomedical entities. In most cases, we observed that the semantic roles of sentence constituents resemble the semantic roles of the entities mentioned therein.

We therefore conclude that the proposed approach bridges the gap between the more co-occurrence based approaches lacking semantic information on the one hand and sophisticated, often computationally expensive semantic approaches on the other hand which are designed to specifically extract only certain types of biomedical relations.
